# Transition of patients with Gaucher disease type 1 from pediatric to adult care: results from two international surveys of patients and health care professionals

**DOI:** 10.3389/fped.2024.1439236

**Published:** 2024-08-27

**Authors:** Karolina M. Stepien, Irena Žnidar, Beata Kieć-Wilk, Angel Jones, Daniela Castillo-García, Magy Abdelwahab, Shoshana Revel-Vilk, Ella Lineham, Derralynn Hughes, Uma Ramaswami, Tanya Collin-Histed

**Affiliations:** ^1^ Adult Inherited Metabolic Diseases, Salford Royal Organization, Northern Care Alliance NHS Foundation Trust, Salford, United Kingdom; ^2^ International Gaucher Alliance (IGA), London, United Kingdom; ^3^ Metabolic Diseases Office, Krakow Specialist Hospital St. John Paul II, Krakow, Poland; ^4^ Unit of Rare Metabolic Diseases, Medical College, Jagiellonian University, Krakow, Poland; ^5^ Department of Pediatrics, Hospital Infantil de México Federico Gómez Instituto Nacional de Salud, México City, México; ^6^ Pediatric Hematology/BMT Unit and Social and Preventive Center KasrAlainy Hospital, Faculty of Medicine, Cairo University Pediatric Hospital, Cairo, Egypt; ^7^ Gaucher Unit, Pediatric Hematology/Oncology Unit, the Eisenberg R&D Authority, Shaare Zedek Medical Center, Jerusalem, Israel; ^8^ Faculty of Medicine, Hebrew University, Jerusalem, Israel; ^9^ Rare Disease Research Partners (RDRP), MPS House, Amersham, United Kingdom; ^10^ Lysosomal Disorders Unit, University College London and Royal Free London NHS Foundation Trust, London, United Kingdom; ^11^ Lysosomal Disorders Unit, Department of Infection, Immunity and Rare Diseases, Royal Free London NHS Foundation Trust, London, United Kingdom

**Keywords:** healthcare transition, Gaucher, child and adolescent health, transition clinic, transfer of care

## Abstract

**Introduction:**

Gaucher disease (GD) is a rare, autosomal recessive lysosomal storage disorder caused by a deficiency in the enzyme glucocerebrosidase. The most common subtype in Europe and the USA, type 1 (GD1), is characterized by fatigue, cytopenia, splenomegaly, hepatomegaly, bone disease, and rarely pulmonary disease. Increased life expectancy brought about by improved treatments has led to new challenges for adolescents and their transition to adult care. Efficient healthcare transition to adult care is essential to manage the long-term age-related complications of the disease.

**Methods:**

This international study consisted of two online surveys: one survey for patients with GD1 and one survey for healthcare professionals (HCPs) involved in treatment of patients with GD1. The aims of this international, multi-center project were to evaluate the current transition process in various countries and to understand the challenges that both HCPs and patients experience.

**Results:**

A total of 45 patients and 26 HCPs took part in the survey, representing 26 countries. Our data showed that a third (11/33) of patients were aware of transition clinics and most stated that the clinic involved patients with metabolic diseases or with GD. Seven patients attended a transition clinic, where most patients (5/7) received an explanation of the transition process. Approximately half of HCPs (46%; 12/26) had a transition clinic coordinator in their healthcare center, and 10 of HCPs had a transition clinic for patients with metabolic diseases in their healthcare center. HCPs reported that transition clinics were comprised of multi-disciplinary teams, with most patients over the age of 18 years old managed by hematology specialists. The main challenges of the transition process reported by HCPs included limited funding, lack of expertise and difficulty coordinating care amongst different specialties.

**Discussion:**

Our study demonstrates the lack of a standardized process, the need to raise awareness of transition clinics amongst patients and the differences between the transition process in different countries. Both patients and HCPs expressed the need for a specialist individual responsible for transition, efficient coordination between pediatricians and adult specialists and for patient visits to the adult center prior to final transition of care.

## 1 Introduction

Gaucher disease (GD) is a rare, autosomal recessive lysosomal storage disorder (LSD) caused by a deficiency of the enzyme glucocerebrosidase, which leads to the accumulation of its substrate, glucosylceramide, in macrophages, preventing their normal function (1). Enlarged macrophages containing undigested glucosylceramide are also known as Gaucher cells and are a pathological hallmark of GD (2). The buildup of Gaucher cells in the spleen, liver, bones, bone marrow, and other tissues cause a progressive loss of organ function, and account for the clinical symptoms associated with the disease (3). The incidence of GD is estimated at 1:40,000–1:60,000 live births and occurs in all ethnicities, although higher incidences are found in Ashkenazi Jews (1:800) (1, 4, 5). The incidence of type 1 GD (GD1) in Europe and North America was reported as between 0.45-22.9/100,000 live births (6).

GD historically has been classified into three main types based upon clinical signs and age of onset: type 1, type 2, and type 3 (7). Although the classification of GD subtype aids clinical management, GD comprises a wide phenotypic spectrum of disease, similar to other LSDs (7). GD1, the most common subtype in US and Europe, is characterized by fatigue, cytopenia, splenomegaly, hepatomegaly, bone disease, ophthalmic abnormalities and on occasion pulmonary disease, dental manifestations, lymphadenopathy and Gaucheroma (8–11). More recent studies have also demonstrated the presence of neurological conditions, including Parkinson's disease, indicating a broader spectrum of disease than previously thought (12, 13). Patients diagnosed with GD1 in childhood generally have more pronounced visceral and bone disease symptoms in comparison with those diagnosed in adulthood. In adulthood, bone disease is the most incapacitating manifestation of GD1, affecting one third of patients (8–11).

Diagnosis of GD is by biochemical testing and the age of GD1 diagnosis varies depending on the population under study. Results from a French Gaucher registry of over 500 patients reported a mean age at diagnosis of 17.4 years, compared to 27.2 years from the international ICGC Gaucher registry (NCT00358943) with over 1,600 patients (14, 15). Biochemical testing identifies deficient glucocerebrosidase activity in peripheral blood leukocytes or other nucleated cells, and genetic testing determines biallelic pathogenic variants in *GBA1*, the gene encoding glucocerebrosidase and is useful for risk prediction, stratification, and counseling (8). Biomarkers like glucosylsphingosine (lyso-Gb1) aid in diagnosis and monitoring disease progression and treatment response Current treatments for GD1 include enzyme replacement therapy (ERT) and substrate reduction therapy (SRT) (16). Potential therapies in clinical trials include several gene therapies (NCT05487599; NCT05324943) and ambroxol, an oral chaperone therapy (NCT01463215), which is mainly studied for neuronopathic GD (type 2 and 3 GD).

Data from the ICGG Gaucher Registry demonstrated that patients diagnosed with GD1 treated with ERT showed improvement of both laboratory and clinical parameters, particularly if treatment was started early (17–21). Although advances in treatment of GD1 have improved quality of life, the disease can remain difficult to manage due to the heterogeneity of clinical presentation and some complex cases require a multi-disciplinary team of specialists to manage and coordinate care (22–26).

It is possible that the increased life expectancy brought about by improved treatments has led to new challenges for adolescents and their transition to adult care, including the requirement of additional specialists such as radiologists, gastroenterologists or dentists if patients develop further long-term manifestations and comorbidities of the disease (27–29). Smooth and coordinated transfer of care from pediatric specialists to adult specialists is essential to manage the long-term age-related complications of the disease and to monitor the compliance and response to available therapies (27). Healthcare transition is defined as the planned, purposeful process of preparing adolescents for adult-centered medical care and is recognized as an important aspect of care for patients with inherited metabolic diseases worldwide (22, 27). Successful transition enables the patient to remain engaged with the healthcare system while progressing towards self-directed management, which requires some readiness of the young adult to make their own healthcare decisions (23, 30). Not all centers have specific transition guidelines (31–33) and there appears to be considerable disparity in transition globally (34–37). A framework for a GD1 transition program is under development. However, well-structured guidelines on transition have been published by the UK's National Institute for Health and Care Excellence (NICE), and encompass a set of general guidelines for transition from children's to adult services (38). These guidelines include recommendations for planning transition and supporting infrastructure, including the implementation of transition clinics. In the UK, transition clinics are prearranged and planned. Regular meetings prior to the transition clinic are an opportunity for both pediatric and adult teams to discuss any outstanding investigations, follow up on any social issues and plan which other specialists involved in a young person's care should be informed about the transfer of care to adult metabolic services.

The aims of this international and multi-center study were to evaluate the current transition process in various countries and to understand the challenges that both HCPs and patients experience. This study was designed by members of the International Working Group on Gaucher Disease (IWGGD), which promotes clinical and basic research into GD, and the International Gaucher Alliance (IGA), a patient led international organization.

## 2 Methods

This international study consisted of two online surveys: one survey for patients with GD1 and one survey for HCPs involved in treatment of patients with GD1, worldwide. The survey for patients with GD1 was open to individuals aged 16 years and over and to caregivers of an individual with GD1. The study was open to patients who were in the process of transitioning to adult services, had already transitioned to adult services or had remained under the same team's care throughout their life in their home countries without transitioning to adult care.

### 2.1 The surveys

Clinical experts who are members of the IWGGD (International Working Group for Gaucher Disease) prepared the surveys in collaboration with the IGA (International Gaucher Alliance) and two patient members of the IWGGD. The surveys were available in English only, were hosted on the Survey Monkey platform, and circulated electronically via email and social media. The local IWGGD representatives were able to translate the questions to those willing to participate. The patient survey included 15 questions aimed at gathering information on the transition process and the associated difficulties and needs from the perspective of patients and carers (see Supplementary Material for full list of questions). The HCP survey included 14 questions aimed at gathering information on the status of the transition process, its organization and the associated difficulties and needs from the perspective of HCPs (see Supplementary Material for full list of questions). The surveys included multiple choice questions, with the possibility to write additional text under the option “Other” when available. The survey was active for two months, from 5th October 2021 to 5th December 2021.

### 2.2 Recruitment

Patients were recruited via social media/newsletter advertisement by the IGA. The invitations contained a link to access the survey platform. In addition, 79 direct email invitations containing the link access were sent to potential participants from 57 IGA member countries. IGA members were asked to forward the invitation and link to HCPs in their country.

### 2.3 Ethical considerations

The study was registered as a service evaluation project with Research & Innovation at Salford Royal Organization (NCA) (registration number 23HIP04). The invitations contained details of the study and participants indicated their consent to take part by clicking the link to the survey. Participants completed the surveys anonymously and no personal data related to age, gender or ethnicity was collected.

### 2.4 Data analysis

Data was extracted from Survey Monkey and descriptive statistical analysis was performed using Microsoft Excel. Respondents were given the option to skip survey questions and not all respondents answered every question. The total number of respondents that answered each question are represented by the denominator.

## 3 Results

### 3.1 Responses

Forty-five responses to the patient survey were received. Of these, one patient only answered the first two questions (age and country) and was excluded from the analysis. A further six left the survey after Question 3 (Do you/your child remain under a specialist care?) and eight left after Question 5 (If “yes” to Q3, are you aware of transition clinic?). Most (*n* = 28) of the 30 remaining patients completed the survey to the end, with one stopping after question 11 and one after question 12.

Twenty-six responses to the HCP survey were received, including 20 complete responses. All six of the HCPs who did not complete left the survey after Question 4 (Do you have transition clinic for metabolic patients in your center?), including 3 who answered yes to this question.

### 3.2 Demographics

**Figure 1:**
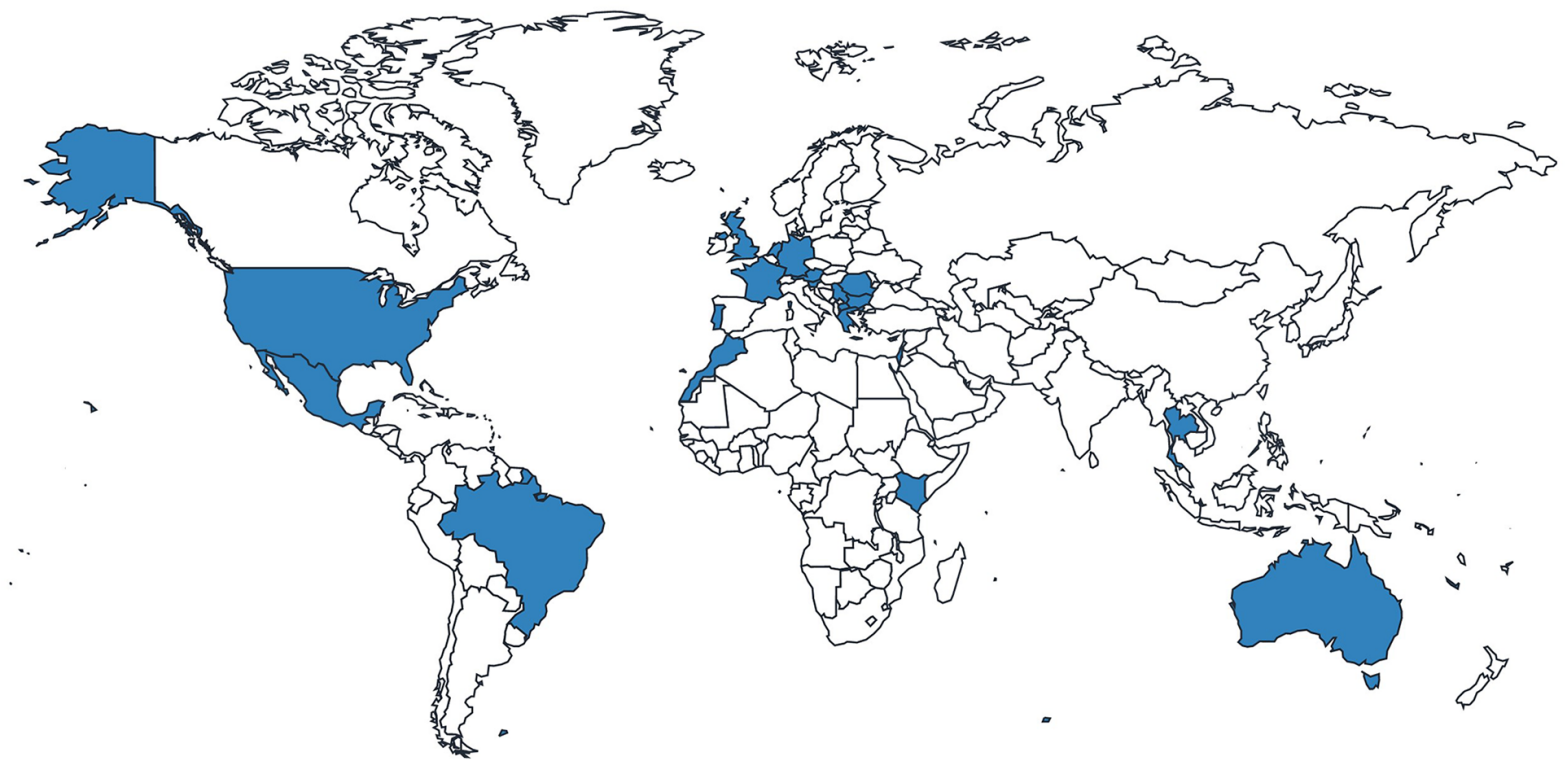
Distribution of responses from patients with Gaucher disease.

Of the 44 GD1 patients included in the analysis, most patients were from the Netherlands, the United Kingdom (UK) and the United States (US) (Figure 1; Table 1). Most patients were over the age of 18 years and were receiving specialist care at the time of the survey (Table 2). Of those patients receiving specialist care, most were cared for by metabolic medicine departments or centers (Table 2). Of the 26 who answered this question, half (*n* = 13) had been treated by a Gaucher disease team in childhood and adulthood.

**Table 1:** Patient country of residence (*n* = 44).

Country	Patients, *n*	Country	Patients, *n*
Netherlands	*n* = 8	Bulgaria	*n* = 1
United Kingdom	*n* = 8	France	*n* = 1
United States	*n* = 6	Germany	*n* = 1
Mexico	*n* = 3	Israel	*n* = 1
Greece	*n* = 2	Kenya	*n* = 1
Romania	*n* = 2	Morocco	*n* = 1
Slovenia	*n* = 2	North Macedonia	*n* = 1
Australia	*n* = 1	Portugal	*n* = 1
Austria	*n* = 1	Serbia	*n* = 1
Brazil	*n* = 1	Thailand	*n* = 1

**Table 2:** Patient characteristics.

Category	*n* (%)
Age	
<18 years	1/44 (2)
≥18 years	42/44 (95)
Not answered	1/44 (2)
Specialist care received	
Yes	37/44 (84)
No	7/44 (16)
Specialist care^a^	
Metabolic medicine	13/33 (39)
Haematology	9/33 (27)
Clinical genetics	5/33 (15)
Internal medicine	4/33 (12)
Other^b^	1/33 (3)
Not answered	1/33 (3)

^a^ Four patients receiving specialist care left the survey before this question.

^b^ Other included: not sure (*n* = 1).

**Figure 2:**
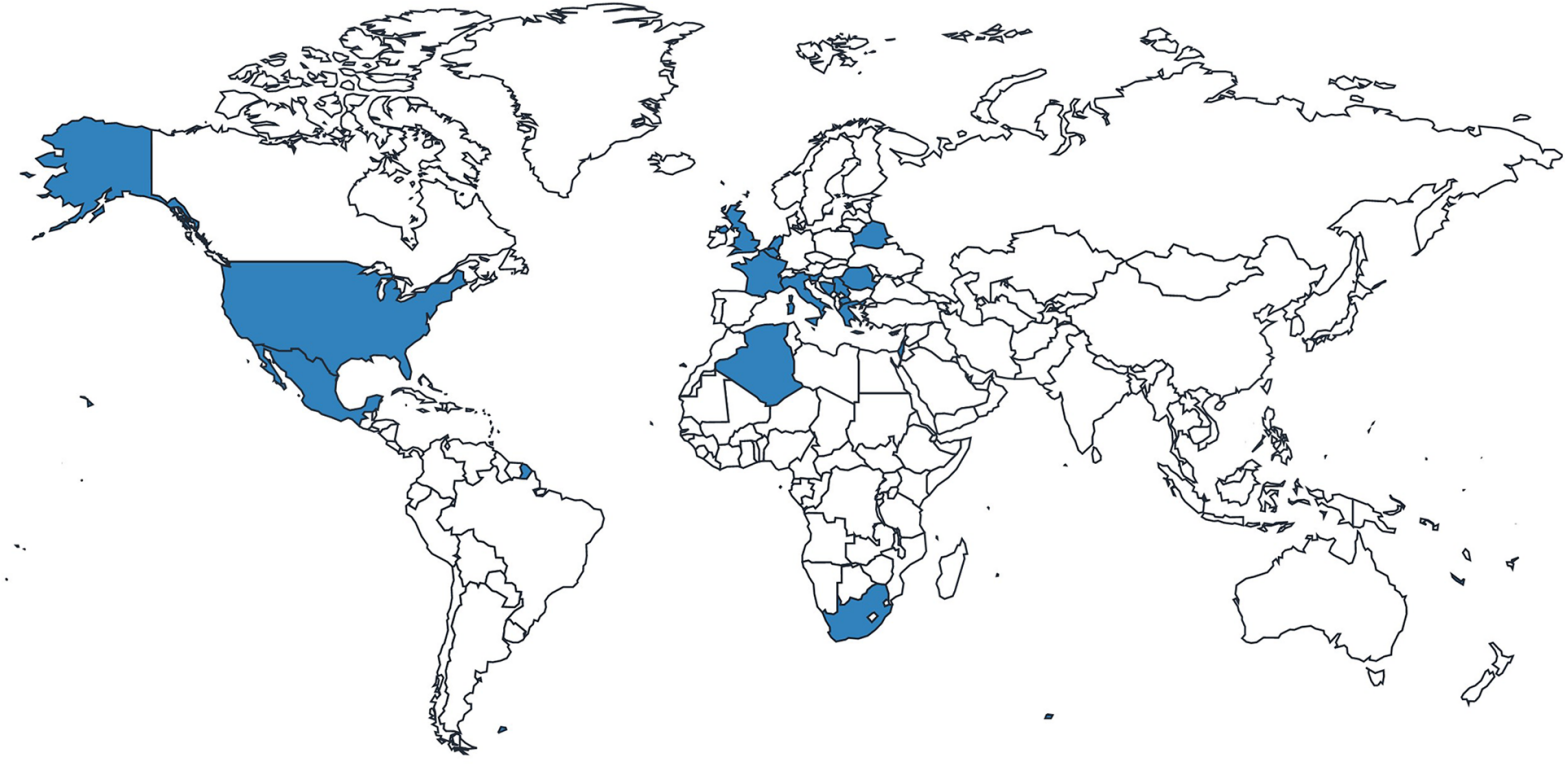
Distribution of responses from HCPs.

Of the 26 HCPs included in the study, almost one-third were from the UK, followed by nearly equal representation of HCPs from other countries worldwide (Figure 2; Table 3). Most specialized in metabolic medicine (Table 4).

**Table 3:** HCP country of work (*n* = 26).

Country	HCPs, *n*	Country	HCPs, *n*
United Kingdom	*n* = 7	Bosnia and Herzegovina	*n* = 1
France	*n* = 2	Greece	*n* = 1
Israel	*n* = 2	Italy	*n* = 1
North Macedonia	*n* = 2	Mexico	*n* = 1
Serbia	*n* = 2	Netherlands	*n* = 1
Algeria	*n* = 1	Romania	*n* = 1
Belarus	*n* = 1	Slovenia	*n* = 1
Belgium	*n* = 1	South Africa	*n* = 1

**Table 4:** HCP characteristics.

Category	*n* (%)
Specialist area	
Metabolic medicine	11/26 (42)
Haematology	5/26 (19)
Clinical genetics	3/26 (12)
Internal medicine	3/26 (12)
General Practitioner	0/26
Other^a^	4/26 (15)

^a^ Other included: paediatric neurology (*n* = 1); gynaecology (*n* = 1); neurology (*n* = 1); paediatric haematology (*n* = 1).

### 3.3 Transition process for patients

Of those patients remaining under specialist care, 33% (11/33) were aware of transition clinics (Table 5). Four patients were from the UK, five were from other European countries, one was from Israel and one was from Mexico (Supplementary Table S1). Of the patients that were not aware of transition clinics, 2/22 did not have a transition clinic available in their respective countries (Australia and Slovenia) and 2/22 patients were diagnosed too late to attend a transition clinic. Most patients who were aware of a transition clinic (8/9) stated that the transition clinic involved patients with GD or metabolic diseases in general.

**Table 5:** Patient experience of transition process.

Category	*n* (%)
Awareness of transition clinic	
Aware of transition clinic	11/33 (33)
Not aware of transition clinic	22/33 (67)
Age patient began attendance at transition clinic^a^	
15–18 years	2/7 (29)
19–22 years	3/7 (43)
23–27 years	0/7 (0)
28–31 years	2/7 (29)
Age of patient at final transfer	
15–18 years	1/7 (14)
19–22 years	2/7 (29)
23–27 years	1/7 (14)
28–31 years	3/7 (43)
Implementation of transition	
Patients received an explanation of the transition process	5/7 (71)
Patients received no explanation of the transition process	2/7 (29)

^a^ Of the 11 patients who were aware of transition clinics, two left the survey before answering any further questions; one patient had not attended a transition clinic and had answered that the transition clinic did not involve metabolic/GD patients; one patient did not answer any of the questions regarding transition.

One patient had been diagnosed as an adult, and while they were aware of transition clinics, they had no personal experience of attending one. Seven patients, from Greece, Israel, Mexico, North Macedonia, Romania, and the UK, provided details of their transition experience (Table 5). Three patients transitioned straight from adolescent care to adult care, two patients attended one transition clinic prior to the final transfer of care (Greece and Romania) and one patient attended four clinics (England). One patient did not say how many clinics they had attended. Patients were aged between 16 and 30 years when they started to attend the transition clinic. Age of final transfer to adult care ranged between 18 and 31 years (Table 5).

Five patients (5/7) received an explanation of the transition process, but none were handed a leaflet about transition, and two received no explanation (Table 5). One patient was asked to complete transition documentation by completing the Ready Steady Go document (39).

#### 3.3.1 Recommendations for improving the transition process

Patients were asked for their suggestions on how to improve the transition process (Table 6). Their recommendations included simplifying the referral process, improving the coordination of transition, including the transfer of patient documents, and making the process more patient friendly, with patients having more say in which specialists they see. Another patient suggested that doctors from the adult care team visit pediatric patients before transition to adult care to enable patients to become familiar with the adult care team and have the opportunity to ask questions about the adult service. One patient remained with the same GD1 specialist from childhood all through to adulthood.

**Table 6:** Patient recommendations for improving the transition process.

“*They need to get the adult doctors to visit the children when they are 16 so they can have a process to get use to going to another clinic, also visits in the adult clinic would help. I would also like to receive a document with all my history from the children clinic and discuss care with the adult doctor. Other problem is that we have no choice as we have to go to hematology, and we do have metabolic and endocrinology clinic and they said no to receiving Gaucher patients as they are all in hematology, which I think should change.*” “*We do not have transition clinics in Bulgaria. One day you're in pediatrics and after you turn 18 you're transitioned to the next clinic. The good thing is that both clinics are 5–10 min apart*. “*I wish there would be more patient friendly transition process.*” “*We need a team of different specialists, not only a haematologist. Some of my paediatric doctors, like my cardiologist is still seeing me but because I specifically ask him to. Even though I have several bone problems I have never had an orthopaedist following my case.*” “*I would hope a process to be simpler and more natural. I think that transition process should be more automatic especially in cases where paediatric patients transition to adult [services] since that transition is inevitable.*”

### 3.4 HCP experience of the transition process

Almost half (12/26) of HCPs had a transition clinic coordinator in their healthcare center (Table 7). In some cases, the transition clinic coordinator was a clinical nurse specialist or a specialist in metabolic or LSDs. Of these HCPs, six were in the UK, with the remaining six of HCPs in other countries. (Belarus, Bosnia and Herzegovina, Mexico, Italy, North Macedonia and Romania).

**Table 7:** HCP experience of transition process.

Category	*n* (%)
Transition clinics	
Presence of transition clinic coordinator	12/26 (46)
Presence of transition clinic for metabolic patients^a^	10/25 (40)
Metabolic transition clinic features^b^	
Presence of transition protocols and guidelines	7/7 (100)
Transition clinic team	
Paediatric team	6/7 (86)
Adult physician and nurse	6/7 (86)
Allied health professionals^c^	5/7 (71)
Subspecialities^d^	3/7 (43)
Common information discussed during transition	
Medical health issues	6/7 (86)
Wellbeing	6/7 (86)
Adult life-related issues^e^	6/7 (86)
Surgical procedures or pre-op assessment	5/7 (71)
Healthy living	5/7 (71)
Consideration of new therapies	4/7 (57)
Other^f^	3/7 (43)
Age patient began attendance at a transition clinic	
14 years	4/7 (57)
15 years	1/7 (14)
16 years	1/7 (14)
18 years	1/7 (14)
Centres with no metabolic transition clinic	
Management of adult patients with GD1	
Metabolic medicine	6/13 (46)
Haematology	7/13 (54)
Clinical genetics	3/13 (23)
Internal medicine	6/13 (46)
Other^g^	3/13 (23)
Main challenges to transition for patients with GD1	
Limited funding	3/13 (23)
Lack of expertise	2/13 (15)
No interest in metabolic medicine	4/13 (31)
Difficulty to coordinate care	5/13 (38)
Patient apprehension	1/13 (8)

^a^ One HCP did not answer this question.

^b^ Three HCPs with metabolic transition clinics left the survey before this question.

^c^ Including physiotherapist, occupational health specialist, dietetics.

^d^ Including orthopaedics, cardiology, neurology.

^e^ Including pregnancy and mental health.

^f^ Other included: dependent on individual (*n* = 1); dependent on consent and patient capacity (*n* = 1); long-term outcomes (*n* = 1).

^g^ Other included: gynaecology (*n* = 1); endocrinology (*n* = 1); oncology (*n* = 1).

#### 3.4.1 Transition clinics for metabolic patients

Ten of 25 HCPs had a transition clinic for metabolic patients in their healthcare center (Table 7; Supplementary Table S1). Two HCPs working in the UK described monthly transition clinics that took place in the pediatric hospital, attended by both pediatric and adult teams, and one HCP working in the Netherlands reported an individualized approach, where harmonization of protocols is standard practice. Physical transition takes place if needed, otherwise the transition is an administrative process. All HCPs (7/7), that had a transition clinic for metabolic patients in their healthcare center had transition protocols or guidelines (Table 7). In the UK, documentation included transition standard operating procedures and Growing Up and Gaining Independence (GUGI), a framework to encourage and support young people to become as independent as they can with their healthcare (40). In the Netherlands, protocols for treatment, diagnosis, follow-up and care pathways were harmonized, and treatment decisions were always made in a multidisciplinary team meeting.

Most HCPs reported the number of transition clinic appointments as a range, between one and four, although the HCP from Bosnia and Herzegovina stated that patients attended six clinics before transition and one for a final report. One HCP explained that the number of clinics attended by patients was dependent on the individual needs of the patient.

HCPs reported that transition clinics were comprised of multi-disciplinary teams, including the pediatric team, adult physician and nurse, and allied health professionals, with additional subspecialities included on an individualized basis (Table 7).

The most common information discussed during transition included medical health issues, wellbeing, and adult life-related issues (Table 7). Patients were between 14 and 18 years old when they started to attend transition clinics (Table 7). Two HCPs explained that age of attendance to transition clinic was dependent on the individual including the level of complex needs and severity of co-morbidities. The age of final transfer to adult care was generally between 16 and 18 years, apart from one center where the range was 16–21 years.

#### 3.4.2 Centers without a metabolic transition clinic

In centers without a metabolic transition clinic, patients with GD over the age of 18 years were mostly managed by hematologists (7/13), internal medicine specialists (6/13) and metabolic specialists (6/13) (Table 7).

The main challenges to provision of a transition service for patients with GD in these centers included limited funding, lack of expertise and interest in adult metabolic medicine in many countries and difficulty coordinating care amongst different specialties. One HCP mentioned patient apprehension as the main challenge for transition (Table 7).

#### 3.4.3 Recommendations for improving the transition process

Suggestions from HCPs included:

• Improved coordination and education of pediatric and adult teams on GD and other hereditary rare diseases with multisystem health problems.
• Standardization of care, guideline development and national approaches.
• Regular review of processes with patients and healthcare professionals to ensure transition is undertaken correctly.
• Visits to adult center for pediatric patients and the management of expectations for both adolescents and parents.
• Empowerment and support of young adults, with an individualized approach to adult care.
• A transition clinic coordinator who has experience within the pediatric service and close teamwork between pediatric and adult care teams.

## 4 Discussion

The results from our multi-center study give an international overview of the status of the transition process from the perspective of both patients with GD1 and HCPs involved in patient care. The majority of patients were cared for by metabolic specialists. The remainder were managed by hematology, genetics or internal medicine departments. This highlights the multi-disciplinary nature of GD, the coordination needed between specialists and the requirement for distinct types of care in the management of the disease. In a recent European study of transition in inherited metabolic diseases, while most centers had an adult metabolic team, only 31% were available to all metabolic conditions (27). This shortage of adult metabolic specialists often leads to fragmented care as patients require monitoring by different specialists, which exacerbates the impact of living with GD1, as patients must repeat information to different providers (27). Indeed, one of the greatest challenges that HCPs reported in this study, was that care of patients with GD1 was spread among different specialties, making it difficult to coordinate their care.

The management of GD is intrinsically challenging due to the phenotypic heterogeneity of the disease (22–26). Management of long-term age-related GD complications is, to a greater degree, complex and requires a smooth and coordinated transfer of care from pediatric specialists to adult specialists (27). Recommendations for the management of pediatric patients focus on the assessment of growth profile and routine neurological examination, including eye movements, while the follow-up of adult patients comprises monitoring of biochemical, hematological, and visceral parameters, in addition to a surveillance of bone disease and assessment of neuropathic pain (22–26). Patients with GD have a diminished health-related quality of life, with poorer outcomes reported in children. Special support should be given to adolescents, particularly during the transition of care to adulthood and a self-directed management. Transition clinics represent, thus, a foundational structure for both pediatric and adult teams to cooperate and effectively support the continuous management of GD tailored to the patients’ developmental maturity and needs (23, 30).

### 4.1 The need for transition coordinators

In this study, only a third of patients were aware of transition clinics. Forty percent of HCPs had a transition clinic for metabolic diseases in general in their center. Patients with GD1 are reviewed in these clinics and none of the centers had a separate transition clinic for them. As patients and HCPs were not matched, these results may reflect the inter and intra-country differences in transition clinic provision, participation bias with HCPs with transition clinics being more likely to complete the survey or a lack of communication on the availability of transition clinics. While recommendations exist on the need for and importance of a named transition coordinator (41), our study suggests they are not available in many centers. In centers that provided metabolic transition clinics, only 70% had a transition clinic coordinator. In a recent European survey, only 31% of inherited metabolic disease centers had a designated transition coordinator, while a French study showed that only 48% of patients with LSD were appointed a transition coordinator (27, 35). A transition clinic coordinator is essential to ensure that adult teams are aware of the management required for rare disorders such as GD1 and are important to guarantee the continuity of care and improvement of outcomes (27).

### 4.2 Adolescent care

While patients first attended transition clinics between the ages of 16–30 years, HCPs reported that attendance usually started at 14–18 years. Young people have different needs to those of children and adults. Some symptoms of GD may also become more pronounced during adolescence. Therefore, starting the transition process in early adolescence allows the patient and parents time to adjust to the changes ahead and may avoid some of the challenges encountered, such as reluctance to attend appointments with an unfamiliar adult team and attachment to pediatricians (30). Appropriate adolescent services prevent young adults with GD from transitioning to adult metabolic services prematurely and being treated as a child in other specialist clinics. Adolescent medicine needs better recognition, particularly in metabolic disorders.

### 4.3 Differences between centres

However, transition must also be flexible and personalized, taking into account the individual needs, cultural differences and circumstances of the patient (42, 43). This flexibility was commented on by the HCPs in this study, particularly in relation to the number of transition clinics offered. Our study showed variability in the number of transition clinics patients attended before final transfer. Patients with fewer health-related problems may only attend one transition clinic, while some other patients with more complex clinical presentation, will need input from other specialties and require more time to transition to adult care. Those patients are likely to remain under the care of several specialties and may attend their own transition clinics.

Although the patients in our study started attending their transition clinic between the ages of 16–30 years, they were 18–31 years old at the final transfer of care to adult services. HCPs reported that final transfer usually occurred at 16–18 years but could be as late as 21 years. The age ranges reported reflect both a personalized approach to individual patients and country differences in the care of adult patients with GD1. The ability of centers to offer flexibility based on the patients’ developmental maturity often depends on the institution, cultural differences, or country regulations. The causes for the delayed transfer of care among patients with GD are not clear but are likely to be related to the availability and readiness of the adult team to take over the care rather than clinical reasons. For example, in Sweden and Italy, patients must transfer by the age of 18 years (44, 45). In the UK, final transfer must also take place before 18 years old as adult patients are not permitted to be hospitalized in pediatric hospitals due to legal restrictions. At 18 years of age, the primary receiver of information changes from the parent or caregiver to the young person (38). In some countries, transition can occur at a younger age. In Oman the transfer age is 13 years, although a recent study of transition readiness recommended that this be increased to 18 years (46).

In this and previous studies, a proportion of adult patients remain under the care of their pediatric team. In a French study of patients with LSD, including some patients with GD1, 24% of patients over the age of 21 were still cared for by pediatric departments (35). A European multi-center study found that 11% of patients remained under pediatric care throughout their lifetime (27). The reasons for patients remaining with pediatric services include a lack of transition organization, lack of disease knowledge in the adult center and refusal to transition by the family or patient (35). In addition, a successful transition process is dependent on standardized operating procedures and adequate financial resources and specific training. The European multi-center survey assessing the challenges associated with transition of patients with inherent metabolic disorders revealed that 90% of HCPs responders reported the absence of financial support for transition programs (27). Noteworthy, one patient in our study wished to remain with the same GD1 specialist from childhood through to adulthood. Patients and parents often get used to the pediatric team and find it difficult to adapt to the changes brought about by transition into adult care. Clear communication has been shown to increase patient and family satisfaction with the transition process (41, 47).

### 4.4 Transition documentation

Of the HCPs surveyed in our study, most stated that their transition clinics had transition protocols or guidelines, although there was a lack of standardization between centers. Transition clinics were comprised of a pediatrician, adult physician and a nurse, along with allied health professionals and subspecialities, reflecting the complexity and heterogenicity of the disease. We found that adult-health related issues such as pregnancy and mental health were amongst the most common issues discussed at transition clinics. Recommendations for patient management defined through a Delphi consensus in Spain include a multi-disciplinary care during pregnancy involving GD specialist, obstetrician, and anesthesiologist. Similarly, experts recommended the involvement of radiologists with experience in GD and orthopedic surgeons for an adequate monitoring of bone disease in patients with prosthetics (22–26). Our study indicates that most patients that attended transition clinics received an explanation of the transition process, although none were given a transition leaflet and only one completed transition support documentation. The absence of standard process and written information on transition may lead to a fear of adult care and an increase in patient and family anxiety (48). An understanding of the challenges related to the transition and long-term follow up is crucial to empower patients on their autonomy and participation in the decision-making process regarding choice of treatment or even frequency of ERT. There is growing evidence that transition programs improve patient outcomes and wellbeing (49). A structured transition program increases patient satisfaction, independence, and perceived health status. A systematic review of transitional care programs in patients with chronic illness or disability aged between 11 and 25 years has shown that the most frequently used strategies in successful programs were specific transition clinics and patient education (50). Initiatives such as the Ready Steady Go Program aim to give patients and their families the knowledge and skills needed to manage their condition into adulthood and have been shown to improve long-term outcomes (39, 51).

### 4.5 Improving the transition process

Patients and HCPs had similar views on how to improve transition through improved coordination and providing opportunities to meet the adult team before transition. Patients wanted a more patient centric process that gave them more say in how their care was managed. HCPs emphasized the need for education, standards, and national approaches in combination with the flexibility to provide individualized care. One of the suggestions is a clinical review of a young adult after the transfer of care to the adult services jointly by pediatric and adult teams. It may empower patients and encourage them to engage with the management of their condition by the new team.

Our findings suggest that the transition process is not well developed in many countries, which may compromise patient care. The main challenges to provision of a transition service for patients with GD in these centers included limited funding, lack of expertise and interest in adult metabolic medicine in many countries and difficulty coordinating care amongst different specialties. One HCP also mentioned patient apprehension as the main challenge for transition.

In the UK, a set of general guidelines for transition from children's to adult services have been published by NICE (38). Within these guidelines, a set of quality standards have also been developed to measure progress and improve the quality-of-care providers are able to deliver. One of the overarching principles of the NICE guidelines was the encouragement of health and social care managers to work together and develop jointly agreed and shared transition protocols, information-sharing protocols and approaches to practice within the UK (38). The lack of harmonization of existing protocols, and inconsistency of outcomes and quality indicators between different countries remain a significant challenge during the transition process (43). The IWGGD have, therefore, developed transition and coordination of care guidelines to standardize care and support the transition process for patients with GD1 (52).

### 4.6 The multidisciplinary team

**Figure 3:**
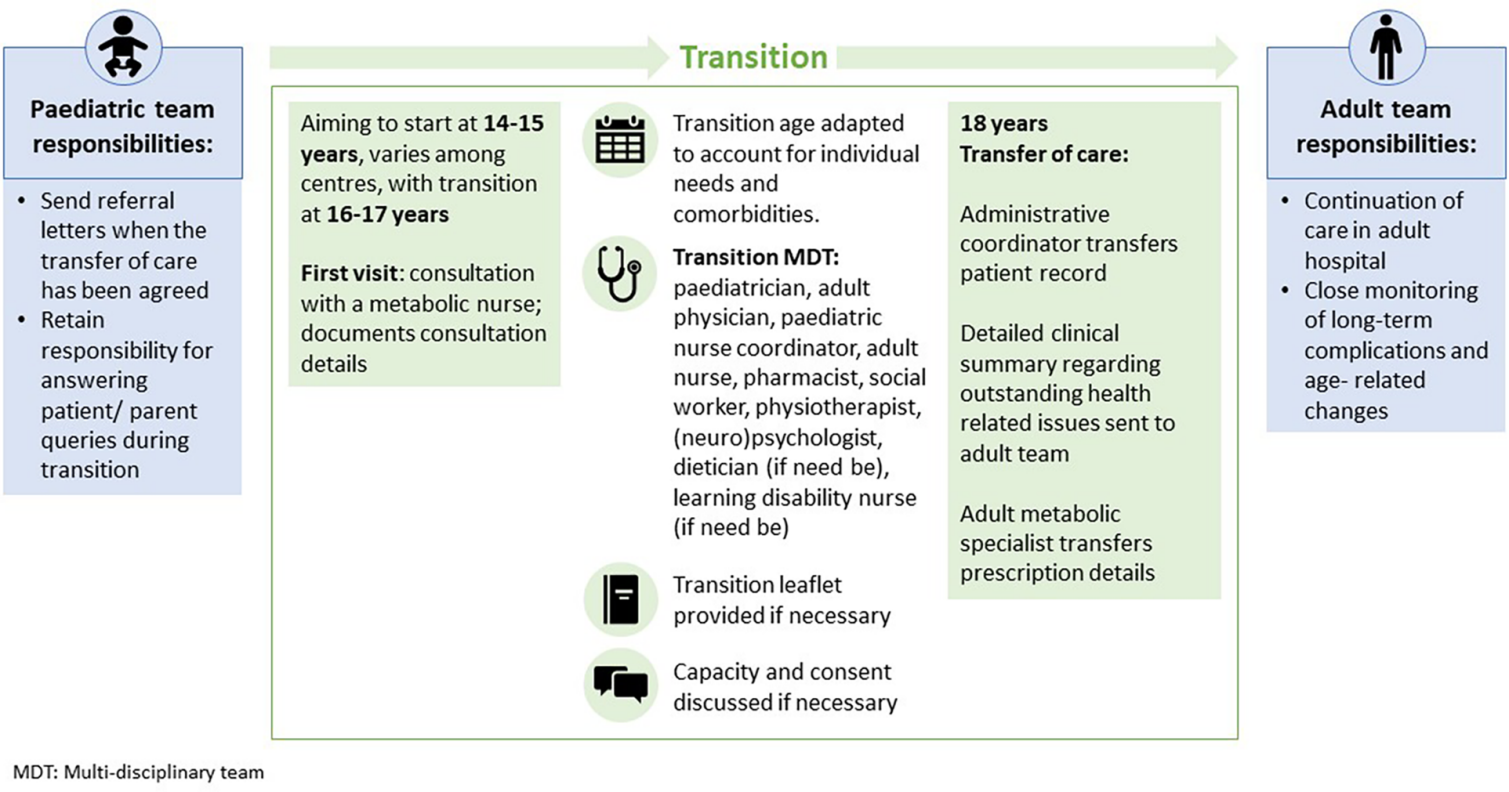
Transition strategy for patients with Gaucher disease.

Transition of patients with GD1 should involve the relevant clinical specialties, psychologists and social workers in the multi-disciplinary team and provide the education and information (e.g., leaflets or apps) necessary to support the patient's independence (Figure 3). Patients are encouraged to understand and manage their disease and acquire the skills and knowledge necessary for self-management, allowing them to act independently. They may attend transition clinics without their parents (52).

Patient organizations can play an important role in supporting the transition process by providing educational resources to help patients understand their condition, treatment, and transition itself, but also in being available for other support needs such as social issues and mental health. It has been recommended that patients are signposted to these services (41). Successful collaboration between clinicians and patient organizations, such as the IWGGD, can lead to the development of guidelines and practices that ensure patients are adequately supported during transition (52).

Well-developed transition guidelines may have potential application in other circumstances where patients need to move clinics, such as when relocating or moving country. There is also potential for new technologies such as Artificial Intelligence to facilitate transition by complementing the role of a transition coordinator; performing tasks such as scheduling, coordinating and reminding about appointments and the date/time of treatment (53).

### 4.7 Limitations of the study

To our knowledge, this is the first study to focus on the transition in GD1 specifically. Our study simultaneously reported the perspectives of patients and HCPs on the transition process worldwide, providing an overview of the current status of the transition process. One limitation of the study was the small number of HCPs surveyed per country. The results may not therefore provide a precise reflection of their clinical practice and we would need to survey a larger sample of HCPs to obtain a thorough picture of the status of international transition provision. To compound to this limitation, the survey was carried out in diverse regions with markedly different healthcare systems, which hinder the understanding and comparison of transition of care of patients diagnosed with GD. In addition, HCPs and patients were not matched so a direct comparison of their perspectives could not be made. Despite an equal opportunity to participate, the results of our survey might not shed a light on the transition of care across all surveyed countries. Half of the respondents (22/44) were mainly from 3 countries (Netherlands, United Kingdom, United States), while the remaining 22 respondents represented 17 surveyed countries, with 1 to 3 respondents each. Another limitation of our study is the reduced number of respondents from Israel, with only one patient and two HCPs participating in the survey. It would be relevant to have a higher participation from Israel to gain a better overview of the standard operating system in transition care in a country with a high prevalence of GD1. Finally, although our survey took place between October and December 2021, we did not assess whether any of the respondents went through the transition of care in the mist of the Covid-19 pandemic or how the disruption of global healthcare affected the transition process, particularly in GD1 centers that had to temporarily halted transition protocols involving multi-disciplinary teams.

## 5 Conclusions

In conclusion, the development of transitional care requires that pediatricians, adolescent medicine specialists and adult specialists work together with patients and GD association groups to manage the needs of patients with GD. An efficient transition process is essential to reduce patient fear and anxiety when moving to adult services. The results of the surveys demonstrate the variability in the transition process between countries, the lack of guidelines for a standardized process and the increasing clinical need for a harmonized transition program among pediatric and adult specialties. GD is one of the commonest rare diseases, with well understood pathophysiology and available therapies. Nevertheless, the implications of transitional care in the long-term follow-up of patients diagnosed with GD have not been previously analysed. As the complications in GD are treatable, it is important to understand the gaps in transition to empower these patients and encourage them to remain under the adult services’ care. Further research, funding and education of adult physicians is required to improve patients’ quality of life and indirectly their compliance to the therapies around the transfer of care.

## Data Availability

The original contributions presented in the study are included in the article/Supplementary Material, further inquiries can be directed to the corresponding author.
