# Supplementary material for: Transition of patients with Gaucher disease type 1 from pediatric to adult care: results from two international surveys of patients and health care professionals
Source: Front Pediatr. 2024 Aug 27;12:1439236. doi: 10.3389/fped.2024.1439236 (PMC11430091; doi:10.3389/fped.2024.1439236)
Supplement: Supplementary file 1 [file Datasheet1.pdf]

SUPPLEMENTARY MATERIAL

1. **Patient Survey**

The questions were discussed and agreed by clinical experts who are members of the International Working Group of Gaucher Disease (IWGGD) in collaboration with the International Gaucher Alliance (IGA). Two members of the IWGGD were patients and made a substantive contribution to the survey development. The survey was designed in Survey Monkey and circulated electronically via email and social media. The authors agree that some patients had no access to the electronic version of the survey. The local IWGGD representatives were able to translate the questions to those willing to participate.

Patient survey

This survey is for adults with Gaucher disease type 1 (GD1), aged 16 years and above who are going through the transition process or have already transitioned to adult services in their home countries. The aims and objectives of the survey are to obtain more information regarding the transition process, the patient's own experience and perspective regarding the process.

1. What is your age?

   Under 18, 18+

2. Which country to do you live in?

   (Open-ended response)

3. Do you or your child remain under a specialist care?

   Yes/No/Any comments

4. If yes to Q3, which specialty:

   - Metabolic medicine
   - Haematology
   - Clinical genetics
   - Internal medicine specialist
   - General practitioner
   - Other (please specify)

5. If ‘yes’ to Q3, are you aware of transition clinic?

   Yes/No/Any comments

6. If 'Yes' to Q5, does it involve metabolic/GD patients in your centre?

   Yes/No

7. If ‘yes’ to Q5, how old were you/your child when you started attending a transition clinic?

   Age 13, 14, 15, 16, Other (please specify)

8. If ‘yes’ to Q5, if you/your child transitioned to adult team’s care, what was the age at the final transfer? Age 15, 16, 17, 18, Other (please specify)

9. If ‘yes’ to Q5, how many transition clinics did you/your child attend before the final transfer of care to the adult services (the last transition clinic)? (Open-Ended Response)

10. If ‘yes’ to Q5, was the transition process explained to you/your child?

    Yes/No/Any comments

11. If ‘yes’ to Q5, were you handed any transition leaflet in the transition clinic?

    Yes/No/Any comments

12. If ‘yes’ to Q5, were you/was your child asked to complete transition documentation in transition clinic? If so, which one?
    - Transition passport

    - Steady Ready Go document

    - Other (please specify)

13. Were you initially treated by paediatric Gaucher disease team and later in adolescence/adulthood by adult Gaucher disease team?

    Yes/No/Any comments

14. Who manages GD patients over the age of 18 years?

    - Metabolic medicine

- Haematology
- Clinical genetics
- Internal medicine specialist
- General practitioner
- Other (please specify)

15. Do you have any suggestions for further improvement of the transition process for GD patients in your centre/country? (Open-ended response)

16. If you are aware of a transition clinic but have not attended, please describe your knowledge below: (Open-Ended Response)

2. **Health care professional (HCP) Survey**

HCP Survey

1. Which country to do you work in?

   (Open-Ended Response)

2. What is your specialty?

   - Metabolic medicine
   - Hematology
   - Clinical genetics
   - Internal medicine specialist
   - General practitioner
   - Other (Free text)

3. Do you have a transition clinic coordinator?

   Yes/No/ Any comments

4. Do No you have a transition clinic for metabolic patients in your centre?

   Yes/No / Any comments

5. If ‘yes’ to Q4, do you have transition protocol/guidelines in your centre?

Yes/No/ Any comments

6. If ‘yes’ to Q4, what is the age at which patients start attending transition clinics?

   Age 13, 14, 15, 16, Other (please specify)

7. If ‘yes’ to Q4, how many transition clinics do patients attend before the final transfer (the last transition clinic)? (Open-ended response)

8. If ‘yes’ to Q4, what is the age of the final transfer of care?

   Age 15, 16, 17, 18, Other (please specify)

9. If ‘yes’ to Q6, is this different for disease types (complex, neurocognitive impairment etc)? Yes/No/ Any comments

10. Who is part of the transition clinic?

    - Paediatrics team
    - Adult physician and nurse
    - Allied health professionals: physiotherapist, occupational health specialist, dietetics etc,
    - Subspecialties - ortho, cardiology etc;
    - Any comments (free text)

11. What information is discussed during transition?

    - Medical health issues
    - Surgical procedures or pre-op assessment
    - Wellbeing
    - Healthy living
    - Considering new therapies
    - Adult life related issues i.e. pregnancy etc
    - Other (please specify)

12. If ‘no’ to Q4, who manages Gaucher patients > 18 years of age in your country?

    - Metabolic medicine
    - Hematology
    - Clinical genetics
    - Internal medicine specialist

- General practitioner
- Other (please specify)

13. If ‘no’ to Q4, what are the main challenges to a transition service for patients with GD patients in your centre?

- Limited funding

- Lack of expertise

- No interest in adult metabolic medicine

- The care of patients with GD is spread among different specialties, so it is difficult to coordinate their care

- Others (please specify)

14. Given that a well-established transition service requires coordination of care, knowledge of rare diseases among paediatric and adult teams, funding, protocols and documentation, do you have any suggestions for further improvement of the transition process for GD patients in your centre/country? (Open-Ended Response)

3. Supplementary Tables

Supplementary Table 1. Awareness and presence of transition clinics

Country	Are GD1 patients aware of transition clinics?		Do HCP's have a metabolic transition clinic at their centre?	
Number of respondents, n	Yes	No	Yes	No
Algeria	–	–	0	1
Australia	0	1	–	–
Austria	Not answered	Not answered	–	–
Belarus	–	–	0	1
Belgium	–	–	0	1
Bosnia and Herzegovina	–	–	1	
Brazil	Not answered	Not answered	–	–
Bulgaria	Not answered	Not answered	–	–
France	1	0	0	1
Germany	0	1	–	–
Greece	1	0	0	1
Israel	1	0	0	2
Italy	–	–	0	1
Kenya	0	1	–	–
Mexico	1	1	1	0
Morocco	Not answered	Not answered	–	–
Netherlands	0	7	1	0
North Macedonia	1	0	1	1
Portugal	0	1	–	–
Romania	1	1	0	1
Serbia	0	1	0	2
Slovenia	1	1	1	
South Africa	–	–	0	1
Thailand	Not answered	Not answered	–	–
United Kingdom	4	2	–	–

Country	Are GD1 patients aware of transition clinics?		Do HCP’s have a metabolic transition clinic at their centre?	
United States	0	5	–	–

– indicates no patient/HCP responded from that country

GD1: Gaucher disease type 1; HCP: health care professional
